# Deciphering the link between salicylic acid signaling and sphingolipid metabolism

**DOI:** 10.3389/fpls.2015.00125

**Published:** 2015-03-09

**Authors:** Diana Sánchez-Rangel, Mariana Rivas-San Vicente, M. Eugenia de la Torre-Hernández, Manuela Nájera-Martínez, Javier Plasencia

**Affiliations:** ^1^Departamento de Bioquímica, Facultad de Química, Universidad Nacional Autónoma de MéxicoMéxico City, México

**Keywords:** salicylic acid, sphingolipid, ceramide, sphingoid bases, sphinganine-analog mycotoxin

## Abstract

The field of plant sphingolipid biology has evolved in recent years. Sphingolipids are abundant in cell membranes, and genetic analyses revealed essential roles for these lipids in plant growth, development, and responses to abiotic and biotic stress. Salicylic acid (SA) is a key signaling molecule that is required for induction of defense-related genes and rapid and localized cell death at the site of pathogen infection (hypersensitive response) during incompatible host–pathogen interactions. Conceivably, while levels of SA rapidly increase upon pathogen infection for defense activation, they must be tightly regulated during plant growth and development in the absence of pathogens. Genetic and biochemical evidence suggest that the sphingolipid intermediates, long-chain sphingoid bases, and ceramides, play a role in regulating SA accumulation in plant cells. However, how signals generated from the perturbation of these key sphingolipid intermediates are transduced into the activation of the SA pathway has long remained to be an interesting open question. At least four types of molecules – MAP kinase 6, reactive oxygen species, free calcium, and nitric oxide – could constitute a mechanistic link between sphingolipid metabolism and SA accumulation and signaling.

## Introduction

Salicylic acid (SA) is a phytohormone involved in local and systemic resistance (Vlot et al., 2009), as well as in the response to abiotic stress, growth, and development (Rivas-San Vicente and Plasencia, 2011). The SA signaling pathway requires a functional NPR1 [nonexpressor of pathogenesis-related (PR) genes 1] protein to relay the signal to the nucleus, where it activates *PR* gene expression (Wang et al., 2005; Kumar, 2014; Seyfferth and Tsuda, 2014). SA biosynthesis occurs either through the phenylalanine (PAL) or isochorismate (ICS) pathway, and the relative contribution of each route varies in different species (Chen et al., 2009b; An and Mou, 2011). SA production is controlled by multiple positive and negative regulators (Janda and Ruelland, 2014). Exciting new research reveals that several sphingolipid intermediates induce SA accumulation and affect disease resistance. The objective of this review is to assess the experimental data that link sphingolipid metabolism with SA accumulation and signaling. Such evidence is mainly derived from (1) the phenotypes of *Arabidopsis* and *Nicotiana* plants in which genes involved in sphingolipid metabolism are mutated or silenced, and (2) the effects of sphinganine analog mycotoxins (SAMs, namely AAL and FB1) on sphingolipid metabolism.

## Sphingolipid Metabolism

**FIGURE 1:**
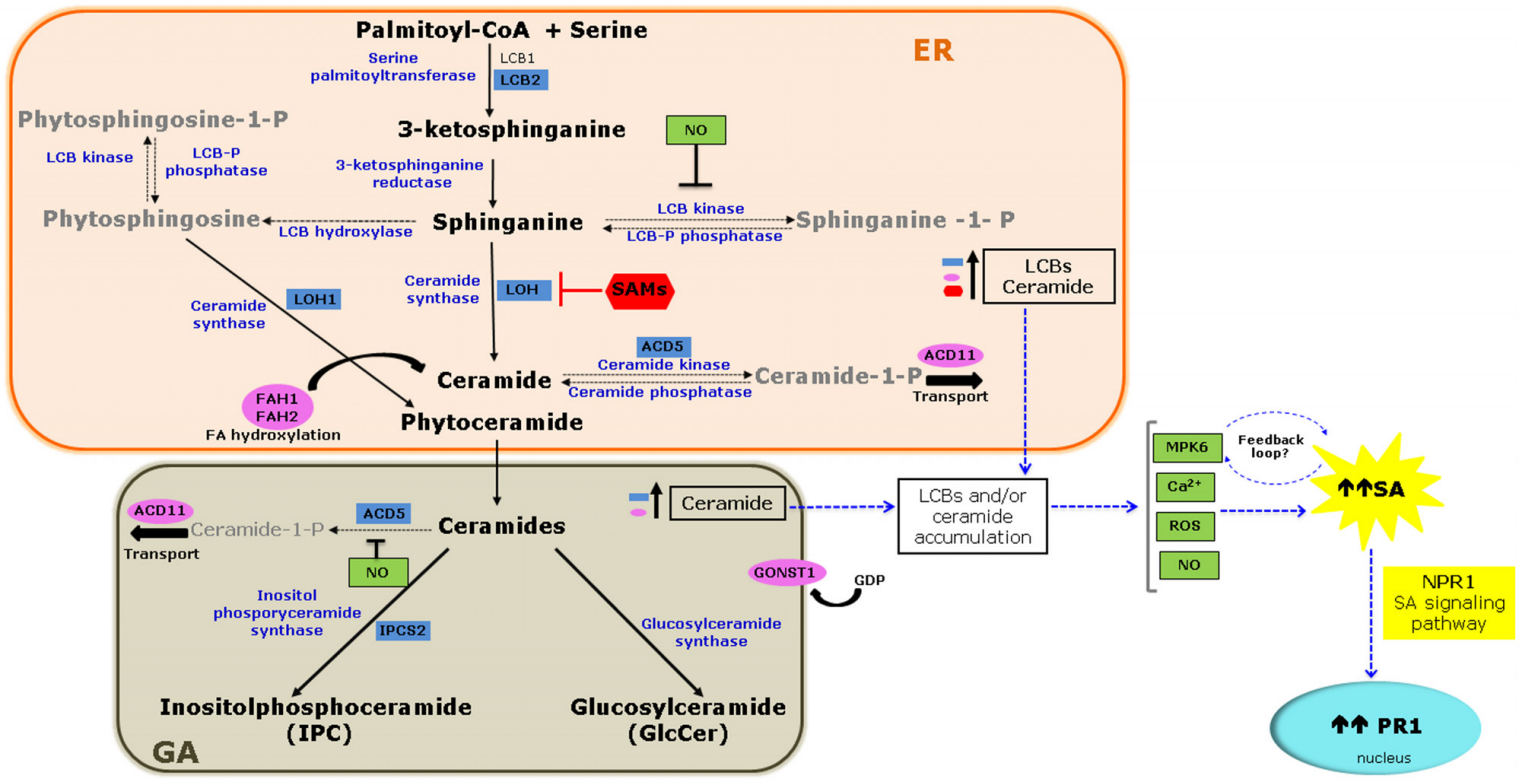
**Molecular links between sphingolipid metabolism and the salicylic acid (SA) signaling pathway.** *De novo* biosynthesis of sphingolipids starts in the endoplasmic reticulum (ER) and ends in the Golgi apparatus (GA) with the biosynthesis of complex sphingolipids. The diagram highlights mutations or silencing events in *Arabidopsis* and *Nicotiana* that result in loss or reduced function of genes involved in sphingolipid metabolism (blue boxes) or sphingolipid modification or transport (pink ovals) and their effect on SA accumulation and/or signaling as a consequence of the accumulation of long-chain base (LCBs), and/or ceramide (see **Table 1**). Also illustrated is the inhibition of the ceramide synthase by sphinganine analog mycotoxin (SAMs; red) that additionally contributes to LCB accumulation and SA pathway activation through pathogenesis-related* (PR1)* gene 1 expression. Several signaling molecules are candidates connecting sphingolipid metabolism and SA signaling (green boxes). MITOGEN-ACTIVATED PROTEIN KINASE 6 (MPK6) may act in a feedback loop between the two pathways. Solid arrows indicate biosynthetic steps, while dashed arrows indicate modification of LCBs and ceramide. Blue arrows denote proposed steps of convergence between the two pathways.

Research in plant sphingolipids has been fostered by the use of novel extraction protocols, followed by mass spectrometry analysis and characterization of *Arabidopsis* mutants. Sphingolipids compose ∼40% of the lipids of the plasma membrane and are also abundant in other endomembranes. Functional genomics of sphingolipid metabolism genes show that these molecules have essential functions in plant growth, development, and stress responses (Chen et al., 2009a; Pata et al., 2010; Berkey et al., 2012). Sphingolipid biosynthesis starts in the endoplasmic reticulum (ER). L-serine is condensed with palmitoyl-CoA to generate a sphingoid long-chain base (LCB) that is reduced and then *N*-acylated to form ceramide. Ceramides are substrates for the production of complex sphingolipids, including inositol phosphorylceramide (IPC), and glucosylceramide. In addition to hydroxylation, LCBs and ceramides can be phosphorylated (**Figure 1**) to yield a wide variety of molecules (Markham et al., 2006).

## Disruption of Sphingolipid Metabolism Through Mutation and Silencing Affects Salicylic Acid Levels

In **Table 1**, we summarize the effects of mutation or silencing of genes involved in sphingolipid biosynthesis or metabolism in *Arabidopsis* ecotypes and *Nicotiana benthamiana*, and highlights the effects of altered SA levels and/or signaling on *PR1* gene expression.

**Table 1:** Association between sphingolipid metabolism and salicylic acid (SA) levels.

Gene name	Gene ID	Gene product	Sphingolipid profile in mutant or silenced plants	SA levels in mutant or silenced plants	*PR1* expression in mutant or silenced plants	Reference
*NbSPT*	NbLCB2 AM902524	Serine palmitoyl transferase (SPT)	50% reduction of trihydroxylated long-chain base (LCBs). Fourfold increase of dihydroxylated LCBs	1.5-fold increase in total SA levels	Constitutive	Rivas-San Vicente et al. (2013)
*AtLOH1*	At3g25540	Ceramide synthase	Higher percentage of ceramides (7%) and glucosylceramides (19%) containing C16 fatty acids. Fivefold elevation of trihyhdroxy-LCBs	SA levels are unaffected	Constitutive; 160-fold raise	Ternes et al. (2011)
*AtFAH1/AtFAH2*	At2g34770/ At4g20870	Fatty acid hydroxylase (FAH)	Fivefold increase of trihydroxylated LCBs; 100-fold reduction of hydroxyceramides; two- to fourfold reduction of phytoglucosylceramides, 10-fold increase of phytoceramides	3.2-fold higher levels of free SA enriched and 4.3-fold increase in SA conjugates	Constitutive	König et al. (2012)
*AtACD5*	At3G21630	Ceramide kinase	Two- to sixfold increase in ceramides and hydroxy-ceramides; no changes in glucosylceramides and LCBs levels	Fourfold increase in free SA and ninefold raise in total SA	Constitutive, but impaired induction.	Greenberg et al. (2000), Bi et al. (2014)
*AtIPCS2*	At2g37940	Inositol –P-ceramide synthase 2	Two- to threefold increase in ceramides and hydroxyceramides. Enriched in trihydroxylated LCBs	Threefold higher levels of free SA and conjugated SA	Constitutive	Wang et al. (2008)
*AtGONST1*	At2g13650	GDP-D-mannose transporter	3.5-fold decrease in the proportion of Hex_1_GIPCs in membranes. Most (75%) of glycosyl inositol phosphorylceramides (GIPCs) found lack hexosylation	Fourfold increase in free SA levels and sixfold increase in total SA	Constitutive; 10-fold raise.	Mortimer et al. (2013)
*AtACD11*	At2g34690	Ceramide-1-phosphate transfer protein	Sevenfold increase in ceramides; threefold rise in hydroxyceramides; twofold increase in GIPC and GlcCer; twofold increase in LCBs and LCB-P	63-fold increase in total SA	Constitutive	Brodersen et al. (2002, 2005), Simanshu et al. (2014)


*Plants with mutated or silenced genes involved in sphingolipid metabolism show alterations in SA levels and/or *PR1* gene expression.*

### Sphingolipid Biosynthesis and Metabolism

#### Long-Chain Base Biosynthesis

Serine palmitoyl transferase (SPT), a heterodimer formed by LCB1 and LCB2 subunits, catalyzes the first reaction in sphingolipid biosynthesis to form LCBs (**Figure 1**; Chen et al., 2009a). The *Arabidopsis* genome contains one gene encoding the LCB1 subunit and two encoding LCB2. Functional studies using mutant and RNAi suppression lines lacking *LCB1* expression, and double mutants lacking both *LCB2* genes, show that sphingolipids are essential for growth and development (Chen et al., 2006; Dietrich et al., 2008). However, it is unknown whether mutations in any of the *LCB* genes affect the SA pathway. A link between SA and sphingolipid metabolism was established through virus-induced gene silencing (VIGS) of the *N. benthamiana* LCB2 subunit. A 20 to 50% reduction in *NbLCB2* transcript level was sufficient to impair growth and leaf and flower development. Compared to control plants, plants with a ∼50% reduction in *NbLCB2* transcripts display elevated SA levels and constitutive *PR1* expression (**Table 1**), and later, show spontaneous cell death in leaves. These silenced plants are also more susceptible to infection by the fungal necrotroph *Alternaria alternata* f. sp. *lycopersici*. LCB composition in silenced plants is altered with lower trihydroxylated LCB and higher dihydroxylated LCB levels than those of control plants (Rivas-San Vicente et al., 2013). These results suggest that disruption of LCB homeostasis is accompanied by elevated SA levels and induction of cell death. However, the identity of the LCB responsible for this phenotype is unknown.

#### Ceramide Biosynthesis

Ceramide synthase catalyzes the condensation of a LCB with a fatty acid-CoA to yield ceramide (**Figure 1**). The *Arabidopsis* genome has three ceramide synthase genes –*LOH1*, *LOH2*, and *LOH3*– and each isoform has a selective preference for the type of acyl-CoA and LCB (dihydroxy- or trihydroxy-LCB; Chen et al., 2009a). Mutants for each gene are viable, and only the *loh1* line has a spontaneous cell death phenotype, which occurs late in development. Although SA levels in this mutant are comparable to those in wild type (WT) plants, *PR1* transcription increased 160-fold (**Table 1**). Furthermore, this mutant exhibits modest changes in sphingolipid content, with a 7 and 19% increase in the proportion of species containing a C16 fatty acid in ceramides and GlcCer, respectively, and a fivefold increase in trihydroxy-LCBs (Ternes et al., 2011). These data narrow-down the identity of the bioactive sphingolipids responsible for triggering cell death to free trihydroxy-LCBs, dihydroxy-LCBs, or ceramide species with a C16 fatty acid.

#### Ceramide Hydroxylation

Ceramide might be hydroxylated in its LCB moiety by a LCB-C4 hydroxylases (SBH) and/or at the fatty acid residues by fatty acid hydroxylases (FAH; Markham et al., 2006). Although double mutants and RNAi suppression lines of *SBH* genes display necrotic lesions in their cotyledons, and constitutively express *PR* genes (Chen et al., 2008), data on SA accumulation and/or signaling is lacking. Conversely, an analysis of double mutants of the two *FAH* genes (*fah1* and *fah2*) demonstrated a link between sphingolipid biosynthesis and SA metabolism. The *fah1/fah2* double mutant displays a 25% reduction in leaf and root growth compared to WT plants, elevated SA levels, and aberrant constitutive *PR1* expression (**Table 1**). Despite elevated SA levels, this mutant lacks a spontaneous cell death phenotype. These plants contain lower levels of ceramides and GlcCer with α-hydroxylated fatty acids, but a 10-fold increase in phytoceramides and a fivefold increase in trihydroxylated LCBs (König et al., 2012). Thus fatty acid hydroxylation of ceramides is required for the biosynthesis of complex sphingolipids and its absence leads to the accumulation of LCBs and ceramides. This elevation activates the SA pathway and supports a link between SA signaling and sphingolipid metabolism.

#### Ceramide Phosphorylation

*ACD5* encodes a 608-amino acid protein with ceramide kinase activity that is located in the ER, Golgi apparatus (GA), and mitochondria (Liang et al., 2003; Bi et al., 2014). In the *Arabidopsis* *acd5* mutant, a glycine residue is replaced with an arginine and the mutant enzyme retains only 10% of the activity of the WT. These mutant plants develop normally for 5 weeks and then display spontaneous leaf lesions, accumulate free, and conjugated SA along with reactive oxygen species (ROS), and constitutively express *PR1* (**Table 1**). Due to reduced ceramide kinase activity, *acd5* plants accumulate ceramides and hydroxyceramides, with a two- to sixfold increase relative to WT plants. Only ceramides containing long-chain fatty acids (C16) accumulate while levels of ceramides with very-long-chain fatty acids (C24 and C26) are not altered (Bi et al., 2014).

The *acd5* cell death phenotype is SA-dependent as it is suppressed in the *acd5/NahG* genotype. *Nah*G encodes a salicylate hydroxylase which converts SA to inert catechol such that these plants do not accumulate SA. Moreover, a functional SA signaling pathway is required because *acd5*/*npr1* double mutants have an attenuated cell death phenotype (Greenberg et al., 2000; Liang et al., 2003; Bi et al., 2014). Mutation in the *ACD5* gene causes an imbalance in the ceramide to ceramide-1-phosphate ratio and ceramide accumulation might activate the SA pathway.

#### Inositol Phosphorylceramide Biosynthesis

Ceramides serve as substrates for the formation of complex sphingolipids (**Figure 1**). Inositol phosphorylceramide-synthase (IPCS) catalyzes the transfer of phosphorylinositol to phytoceramide to yield IPC. The *Arabidopsis* genome contains three functional *IPCS* genes: *IPCS1*, *IPCS2,* and *IPCS3*. *AtIPCS2* is expressed at higher levels than the other two genes in all organs tested (Mina et al., 2010), and the protein localizes to the *trans*-Golgi network (Wang et al., 2008).

The phenotype associated with *IPCS2* loss of function is only discernible in transgenic plants expressing the resistance gene *RPW8* (*Resistance to powdery mildew*). These plants exhibit spontaneous cell death and were thus named *ehr1* (*enhancing RPW8-mediated hypersensitive response cell death*). *Arabidopsis* *RPW8* confers broad-spectrum resistance to powdery mildew, and the *ipcs2* mutation in *RPW8* transgenic plants increased sensitivity to fungal infection. The *ipcs2* mutant lines are only 30% the size of the parental line at maturity and exhibit regions of spontaneous cell death. These plants accumulate both free SA and SA conjugates and constitutively express *PR1* (**Table 1**). Spontaneous cell death and constitutive *PR1* expression depend on SA, since both traits are abolished when the *NahG* gene or the *pad4* mutation is introduced. PAD4 is an upstream regulator of the SA signaling pathway. Again, the precise identity of the sphingolipid molecule responsible for this phenotype remains unidentified, because these mutant plants show increased levels of both ceramides and LCBs (Wang et al., 2008).

### Sphingolipid Modification

Complex sphingolipids, such as glycosyl IPC (GIPC), are the most abundant lipids in plant cell membranes (Markham et al., 2006). Glycosylation of the inositol head group occurs in the GA. Monosaccharides such as hexuronic acid, galactose, mannose, and arabinose can be attached to GIPC (Buré et al., 2011). Guanidine diphosphate (GDP) sugars serve as donors for glycosylation and are transported into the GA. GONST1 (GOLGI-LOCALIZED NUCLEOTIDE SUGAR TRANSPORTER) belongs to a family of nucleotide sugar transporters and stimulates GDP-mannose transport (Baldwin et al., 2001). Knock-out of *GONST1* causes severe dwarfing, poor seed set, formation of spontaneous necrotic lesions on the leaves, accumulation of free and conjugated SA, and constitutive *PR1* expression (**Table 1**). Overexpression of *NahG* in the *gonst1* background diminishes SA levels and the number of necrotic lesions, and partially alleviates the growth defect. These data suggest that the ability to accumulate SA is partly responsible for the *gonst1* phenotype. The *gonst1* plants do not differ from WT plants in ceramide or LCB content, but do exhibit changes in sphingolipid sugar decoration; the proportion of Hex_1_GIPCs in membranes isolated from *gonst1* is 25%, compared to 90% in the WT (Mortimer et al., 2013).

### Sphingolipid Transport

*ACD11* encodes a protein homologous to a mammalian glycolipid transfer protein (GLTP) with no predicted transmembrane domains or localization motifs. It was initially characterized as a sphingosine transporter (Brodersen et al., 2002), but a recent study showed that this protein contains a lipid recognition center. ACD11 selectively binds to ceramide-1-phosphate (C1P) and phyto-C1P, but not to related plant sphingolipids such as ceramides, GlcCers, GIPCs, and LCBs (Simanshu et al., 2014).

*Arabidopsis* *acd11* mutant plants show an accelerated cell death phenotype early in development, characterized by ROS generation, necrotic lesions, and constitutive expression of senescence- and defense-related genes (Brodersen et al., 2002). They also accumulate SA and display constitutive *PR1* expression (**Table 1**). Since the cell death phenotype is suppressed in transgenic *NahG* lines that do not accumulate SA, cell death is SA-dependent. Cell death is also blocked by mutations in *PAD4* and *EDS1*, which are upstream regulators of the SA response (Brodersen et al., 2002). Other mutations in key components of the SA biosynthesis and signaling pathway, such as *SID2* and *EDS5,* also diminish SA accumulation in the *acd11* mutant (Brodersen et al., 2005). *SID2* encodes an ICS synthase, suggesting that SA accumulation is partly responsible for the observed phenotype, and ICS precursors might trigger cell death. The *acd11/eds5* mutant exhibits constitutive *PR1* expression and a similar cell death phenotype as the *acd11* mutants, but limited SA accumulation. *EDS5* encodes an extrusion-like transporter involved in SA export from chloroplasts (Serrano et al., 2013). Exogenous application of the SA analog BTH to *acd11/NahG* and* acd11/sid2* plants restores cell death and induces ceramide accumulation, reinforcing SA role in the signaling pathway that results in this phenotype (Brodersen et al., 2002, 2005).

Both LCBs and ceramides could contribute to the *acd11* mutant phenotype, as levels of these sphingolipid intermediates are elevated in these plants. Because of the functional association between sphingolipid metabolism and SA biosynthesis, a feedback loop might regulate these two pathways (Simanshu et al., 2014).

## SA Levels are Affected by the Action of Sphinganine Analog Mycotoxins on Sphingolipid Metabolism

SAMs share structural similarity with LCBs and inhibit ceramide synthase activity (**Figure 1**) causing LCB levels to increase (Wang et al., 1991; Abbas et al., 1994). The best-characterized SAMs are the AAL-toxin produced by *A. alternata* f. sp. *lyco-persici*, a tomato foliar pathogen, and fumonisin B1 (FB1), produced by *Fusarium verticillioides*, a causal agent of various diseases in maize. Although the hosts and type of diseases caused by these fungi are quite different, genetic evidence supports a role for SAMs in virulence of these fungal pathogens (Sánchez-Rangel and Plasencia, 2010).

### Effects of SAMs in *Arabidopsis* Genotypes and Hormonal Crosstalk

Fumonisin B1 causes LCB accumulation in several plant species (Abbas et al., 1994; de la Torre-Hernandez et al., 2010), and in *Arabidopsis*, a 72-h treatment with 10 μM FB1 triggers a 100- to 7000-fold increase in LCBs concentration (Saucedo-García et al., 2011). This dose also triggers lesions reminiscent of those formed in the pathogen-induced HR, accompanied by callose deposition, ROS and camalexin production, and expression of *PR-1*, *PR-2*, and *PR-5* in *Arabidopsis* leaves (Stone et al., 2000). Very low doses (70 nM) of FB1 cause DNA fragmentation and cell death in *Arabidopsis* protoplasts. Because protoplasts from mutant genotypes defective in SA, jasmonic acid (JA), and ethylene (ET) accumulation or signaling are more tolerant to the toxin, it was concluded that all three signaling pathways are required for cell death caused by FB1 (Asai et al., 2000). However, when 50 μM FB1 was infiltrated into rosette leaves of *dde2*, *ein2*, *pad4*, and *sid2* single mutants (defective in JA, ET, SA, and SA signaling pathways, respectively) and the corresponding quadruple mutant, enhanced tolerance to FB1 was not observed (Igarashi et al., 2013). The cell type tested – protoplasts vs. intact leaves – and the ∼700-fold difference in FB1 dose might account for the observed discrepancies.

From the above data, it is clear that FB1 activates the SA pathway, but also other routes that are antagonistic to SA signaling, thus complicating results interpretation. For instance, *Arabidopsis* possesses five ET receptors (ETR1, ETR2, EIN4, ERS1, and ERS2) and genetic studies show that, in absence of ET, the receptors positively regulate CTR1, which acts as a negative regulator of the ET signaling pathway (Ju et al., 2012). FB1 has contrasting effects in mutants of the five ET receptors; while the *etr1-1* mutant shows hypersensitivity to FB1, the *ein4-1* mutant displays diminished cell death and the other three mutants respond similarly to the WT. These results suggest that ET receptors have distinct roles in toxin sensitivity leading to the HR. While ET induces cell death through EIN4, perception of this phytohormone by ETR1 inhibits cell death. Because ET represses *PR1* transcription, mutations in genes encoding ET receptors increase the expression of SA-inducible genes; for instance the *ers1* and the *ein4* mutants display a 29-fold and 115-fold rise in *PR1* expression, respectively (Plett et al., 2009).

## Deciphering the Link Between Sphingolipid Metabolism and the SA Pathway

Since both, LCBs and ceramides, serve as signaling molecules in the activation of defense-related PCD (Berkey et al., 2012), it is reasonable to hypothesize that SA acts as an intermediate in this pathway. So far, evidence provided by mutants in sphingolipid biosynthesis and by experiments with SAMs and exogenous LCBs/ceramides, suggests that sphingolipid intermediates act upstream of SA. Expression of *NahG* or negative regulators of the SA pathway in sphingolipid mutants confirms that this phytohormone is required for the cell death phenotype. However, the remaining question is how perturbations in levels of LCBs/ceramides are perceived to induce the SA biosynthesis pathway? Several signaling molecules, upstream and/or downstream of sphingolipid intermediates, are candidates to connect these two pathways (**Figure 1**), will be described briefly.

Both, FB1 and LCBs activate MITOGEN-ACTIVATED PROTEIN KINASE 6 (MPK6) within minutes after the infiltration of *Arabidopsis* rosette leaves. Moreover, *mpk6* mutant seedlings show reduced cell death when exposed to 10 μM FB1, suggesting that MPK6 is a transducer in the pathway leading to LCB-induced PCD in *Arabidopsi*s (Saucedo-García et al., 2011). Although MPK6 was characterized as the ortholog of the tobacco SA-induced protein kinase (SIPK), it is rapidly activated by several microbial elicitors (Nühse et al., 2000) with a similar kinetics as with FB1 and LCBs.

Reactive oxygen species elevation is a common feature displayed by several mutants in sphingolipid biosynthesis that show an enhanced cell death phenotype and SA accumulation. Molecules such as hydrogen peroxide (H_2_O_2_) and superoxide anion (O_2_^⋅-^) mediate a variety of cellular responses. In *Arabidopsis*, 10 μM FB1 causes an elevation of *PAL* transcript and activity, which results in a fourfold increase in total SA. This elevation depends on ROS, as inhibitors that disrupt ROS production prevent this response (Xing et al., 2013). Moreover, exogenous LCBs induce ROS production in leaves of *Arabidopsis* seedlings (Shi et al., 2007). Although data on LCB accumulation is lacking in these reports, FB1 biological activity suggests that LCBs elevation mediate ROS generation.

Another hypothesis is that free calcium levels change in response to a sphingolipid imbalance and transduce a signal. In tobacco BY2 cells, exogenous addition of dihydroxy-LCB causes an immediate (∼1 min) dose-dependent elevation of cellular free calcium concentration in the cytosol and 10 min later in the nucleus, followed by H_2_O_2_ accumulation and cell death (Lachaud et al., 2010, 2011). Calcium also regulates the expression of the SA biosynthesis gene *ICS1* through the Ca^2+^/calmodulin-binding transcription factor CBP60g (Wang et al., 2009; Zhang et al., 2010). Another Ca^2+^/calmodulin-binding transcription factor, AtSR1, is a negative regulator of SA signaling, as it controls *EDS1* expression (Du et al., 2009). Because calcium controls SA levels, free cytosolic Ca^2+^ could link sphingolipid metabolism with the SA pathway. Testing the susceptibility of *atsr1* mutants when challenged to an imbalance of sphingolipids levels through exogenous addition of FB1 or LCBs might shed some light on this question.

Finally, nitric oxide (NO), a universal transducer molecule, might play a role in linking sphingolipids and the SA pathway. In *Taxus* sp. cell cultures, a fungal-produced sphingolipid induces rapid and dose-dependent NO production, and because this molecule is a redox regulator of the NPR1/TGA1 system, it promotes NPR1 translocation into the nucleus (Wang et al., 2007; Lindermayr et al., 2010; Guillas et al., 2013). NO might also act upstream of sphingolipids intermediates. Exposure of *Arabidopsis* plants to cold induce NO production that down-regulates the synthesis of phytosphingosine-phosphate and ceramide-phosphate. In the *nia1/nia2* nitrate reductase mutant, impaired in NO biosynthesis, such suppression does not occur (Cantrel et al., 2011). Thus NO could participate in the fine-tuning of the balance between certain sphingolipids and their phosphorylated derivatives.

Studies of the phenotypes of *Arabidopsis* mutants in sphingolipid metabolism suggest that imbalance of LCBs and/or ceramides levels activate the SA pathway. However, further research is needed to determine the causality of this relationship and to identify the upstream signal transduction molecule(s) responsible for activating the SA pathway. Additional comparisons of the effects of FB1, LCBs, and ceramides on MPK, ROS, calcium, and NO signaling in relevant *Arabidopsis* WT and mutants will reveal the main players in this complex interaction between the sphingolipid and SA signaling pathways.
